# Exercising calf muscle 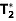 changes correlate with pH, PCr recovery and maximum oxidative phosphorylation

**DOI:** 10.1002/nbm.3092

**Published:** 2014-03-09

**Authors:** Albrecht Ingo Schmid, Kiril Schewzow, Georg Bernd Fiedler, Sigrun Goluch, Elmar Laistler, Michael Wolzt, Ewald Moser, Martin Meyerspeer

**Affiliations:** a Center for Medical Physics and Biomedical Engineering, Medical University of ViennaWähringer Gürtel 18-20, 1090, Wien, Austria; b MR Centre of Excellence, Medical University of ViennaLazarettgasee 14, 1090, Wien, Austria; b Department of Clinical Pharmacology, Medical University of ViennaWähringer Gürtel 18-20, 1090, Wien, Austria

**Keywords:** ^31^P MRS, skeletal muscle, T^*^_2_, exercise, plantar flexion, energy metabolism, pH, 7 Tesla

## Abstract

Skeletal muscle metabolism is impaired in disorders like diabetes mellitus or peripheral vascular disease. The skeletal muscle echo planar imaging (EPI) signal (*S*_EPI_) and its relation to energy metabolism are still debated.

Localised 31P MRS and *S*_EPI_ data from gastrocnemius medialis of 19 healthy subjects were combined in one scanning session to study direct relationships between phosphocreatine (PCr), pH kinetics and parameters of 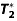 time courses. Dynamic spectroscopy (semi-LASER) and EPI were performed immediately before, during and after 5 min of plantar flexions. Data were acquired in a 7 T MR scanner equipped with a custom-built ergometer and a dedicated ^31^P/^1^H radio frequency (RF) coil array.

Using a form-fitted multi-channel ^31^P/^1^H coil array resulted in high signal-to-noise ratio (SNR). PCr and pH in the gastrocnemius medialis muscle were quantified from each ^31^P spectrum, acquired every 6 s. During exercise, *S*_EPI_(*t*) was found to be a linear function of tissue pH(*t*) (cross-correlation *r* = –0.85 ± 0.07). Strong Pearson's correlations were observed between post exercise time-to-peak (TTP) of *S*_EPI_ and (a) the time constant of PCr recovery *τ*_PCr recovery_ (*r* = 0.89, *p* < 10^− 6^), (b) maximum oxidative phosphorylation using the linear model, *Q*_max, lin_ (*r* = 0.65, *p* = 0.002), the adenosine-diphosphate-driven model, *Q*_max,ADP_ (*r* = 0.73, *p* = 0.0002) and (c) end exercise pH (*r* = 0.60, *p* = 0.005).

Based on combined accurately localised 31P MRS and 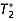 weighted MRI, both with high temporal resolution, strong correlations of the skeletal muscle *S*_EPI_ during exercise and tissue pH time courses and of post exercise *S*_EPI_ and parameters of energy metabolism were observed. In conclusion, a tight coupling between skeletal muscle metabolic activity and tissue 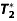 signal weighting, probably induced by osmotically driven water shift, exists and can be measured non-invasively, using NMR at 7 T.

## INTRODUCTION

Skeletal muscle is the main contributor to energy expenditure of the human body and an important target of insulin, which stimulates myocellular nutrient uptake and storage 1. Studying the metabolic and vascular state of skeletal muscle can therefore play a key role in aiding better understanding of diseases like diabetes mellitus and its complications, peripheral artery disease or other cardio-vascular disorders. In this study, dynamic ^31^P MRS was used to investigate exercising skeletal muscle energy metabolism combined with 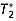 weighted ^1^H MRI, sensitive to tissue water shift and blood oxygenation 2,3, within one measurement session.

Numerous studies have been published using ^31^P MRS techniques, ranging from a more technical focus to basic physiology and various diseases in humans 2,4. In particular, time-resolved *in vivo* concentrations of phosphorylated creatine (PCr), inorganic phosphate (P_i_) and intracellular pH can be quantified. From their kinetics, the adenosine triphosphate (ATP) turnover can be inferred and maximal mitochondrial output *Q*_max_ 5–7 can be derived from PCr recovery rate constants after exercise-induced depletion.

This information is very specific, as ATP turnover is acquired dynamically *in situ*, i.e. directly in the working muscle during and after exercise.

Especially during recovery from exercise or ischaemia, ^31^P MRS data are often interpreted as a measure of mitochondrial capacity or fitness. The underlying assumption is that potential systemic limitations — cardiac output, arterial and venous flow, tissue perfusion and oxygenation — can be ignored and that mitochondrial function is the rate-limiting factor. By combining ^31^P MRS and near-infrared spectroscopy data in patients with peripheral vascular disease, it has been shown that this is not necessarily the case 8. Also, in healthy volunteers 9 and in different types of myositis, capillary perfusion was suggested to limit recovery from exercise 10.

Compared with high-energy phosphate concentrations obtained from ^31^P spectra, alterations in echo planar imaging (EPI) signal intensity (*S*_EPI_) are not so straightforward to interpret 3,11,12. Several effects influence the skeletal muscle EPI signal simultaneously, such as changes in *T*_2_, and 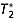, which result from alterations in capillary blood oxygenation, volume and most prominently tissue water distribution. Osmotic effects of altered metabolite and acid-base equilibrium also contribute to alterations in water content. Both higher water content and blood oxygenation increase 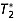 13,12. As summarised by Prompers *et al*. 2, increased metabolic activity such as PCr breakdown into P_i_ and Cr or glycogenolysis into lactate results in accumulation of metabolites to which the cell membrane is relatively impermeable. The accompanying osmotic pressure leads to water shift between intra- and extracellular compartments and hence to observable *T*_2_ changes, first reported by Fleckenstein *et al*. 14. On top of this, effects of altered tissue volume and the blood oxygen level dependent (BOLD) effect contribute to the observed signal intensity. Unfortunately, the relative contributions are not easy to separate and depend on fibre type, exercise duration and intensity 15,16,3,17. When using relatively long *T*_R_, *T*_1_ and flow effects can be neglected.

Recently, a model for a more quantitative analysis of skeletal muscle EPI signal (*S*_EPI_) data was developed 18. A motivation for the present study is to contribute to the interpretation of the muscle *S*_EPI_ effect by exploring the combination of this model with recent improvements of specificity and sensitivity in localised ^31^P MRS at ultra-high field. Even though the model was designed for ischemia-reperfusion *S*_EPI_ data initially, the time courses in exercise recovery share the same features.

In this study, we combined mechanical force measurements, localised ^31^P spectroscopy and functional ^1^H MRI within the same region of interest and high temporal resolution. The benefits of high SNR from 7 T static magnetic field 19 and a dedicated form-fitted multi-channel radio frequency (RF) coil 20 allow for acquisition of dynamic muscle-specific MRS data using accurate localisation with classical excitation and adiabatic selective refocusing (semi-LASER) 21,22 at high temporal resolution (i.e. 6 s). The SNR with this set-up is comparable to that using 3 T non-localised ^31^P spectroscopy with a single loop coil 23 and allows us to focus on one particular muscle only. With *B*_1_ (RF field) localisation only, some signal from other muscles will always contribute, thereby resulting in a mixture of at least two metabolic pools. In particular, the P_i_ resonance is known to broaden or even split, possibly due to physiological 24,25 or partial volume effects 26. Also, the line width is typically lower in smaller voxels. Given sufficient SNR 23, accurately localised spectroscopy can therefore improve data interpretation by reducing ambiguity. In EPI, in addition to the SNR gain by higher magnetic field strengths, the higher sensitivity of the MRI signal to susceptibility at 7 T and generally shorter *T*_2_ increase the contrast in 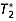 weighted *S*_EPI_ 27,19.

## EXPERIMENTAL

The protocol was approved by the local ethics committee and was in accordance with the Declaration of Helsinki. 21 healthy subjects were recruited; one female was measured twice.

### Data acquisition

Subjects (10 men, 11 women, age 20–30 years, body mass index (BMI) 18.7–25.2 kg/m^2^) were measured on a 7 T scanner (Magnetom 7 T, Siemens Medical Solutions, Erlangen Germany) employing a custom-built ergometer, designed for plantar flexions inside the scanner. An in-house built ^31^P/^1^H transceive coil, shaped to a half cylinder of diameter *d* = 14 cm form-fitted to the human calf, was used for imaging and spectroscopy. The ^1^H channel consisted of two array elements and the ^31^P channel of three 20. The lengths of the ^31^P and ^1^H elements were *l* = 10 cm and *l* = 12.5 cm, respectively.

After instructing and positioning the subject in the ergometer, which was placed on the bed of the MR scanner, maximum voluntary contraction force (MVC) was measured by repeatedly pushing against the blocked ergometer pedal. The resistance of the pedal was then set to 30 % of individual MVC.

First, axial gradient echo images (30 slices, field of view = 160 mm × 160 mm, slice thickness = 4.8 mm, matrix size = 256 × 256, *T*_E_ = 4.5 ms, *T*_R_ = 570 ms) were acquired as anatomical reference and used for region of interest (ROI) placement.

The exercise experiment started 30 min after arrival on site and consisted of two blocks. Each block started with necessary adjustments, including shimming, and required approximately 15 min. The delay also served for standardisation, i.e. volunteers were in a relaxed, resting state. This was followed by 2 min of baseline measurements at rest, 5 min exercise and 20 min recovery for EPI imaging or 7 min of recovery for localised ^31^P MRS. *S*_EPI_ data were always acquired before the ^31^P measurements. This design was chosen because the *S*_EPI_ signal can remain elevated for up to 25 min before returning to pre exercise levels 28,18,29. It allowed for a stable *S*_EPI_ baseline and for ∼ 40 min of recovery before the ^31^P MRS acquisition during the second exercise block. PCr and pH values, on the other hand, are known to return to baseline much faster.

Echo planar imaging (measurement block one) was performed with a repetition time of *T*_R_ = 6 s, *T*_E_ ≈ 20 ms and 270 repetitions, lasting for 27 min. One 6 mm slice with a matrix size of 128 × 128 and an in-plane voxel size between 1.25 and 1.5 mm — depending on calf cross-section — was acquired. Localised ^31^P spectra (measurement block two) were acquired using a single-voxel semi-LASER sequence 21, selecting the maximum double-oblique cuboid volume that fitted into each subject's medial gastrocnemius muscle. It was verified on multiple slices that contributions from the adjacent soleus muscle were not included. The average volume was 57 × 19 × 36 mm^3^ (or 35.7 ± 7.6 cm^3^), while the *T_R_* was 6 s. The RF power was adjusted by varying the system reference voltage to maximise signal from the volume of interest (VOI), as described in 21. The shortest possible echo time was selected, limited by pulse duration and transmit voltage, i.e. *T*_E_ = 24 ms in most subjects, 25 ms in four subjects and 26 ms in one subject. Data were acquired for 14 min.

Between minutes 2 and 7 of each block, the volunteers were asked to perform two plantar flexions every *T*_R_ = 6 s during the inactive periods of the pulse sequence, acoustically triggered by gradient sound.

### Data processing and quantification

The EPI signal was quantified from a ROI in gastrocnemius medialis, excluding large vessels, drawn on the image averaged over the recovery period. The ROIs were applied to the image time series and the signals were integrated for each time point. The resulting time courses were normalised to the median of the first 20 scans, i.e. the baseline during rest. Muscle cross-sectional areas were calculated from the ROIs. Image registration of the EPI data was investigated but not included in the analysis, because misalignments, when present, were too small so that the registration would not improve data quality.

The *S*_EPI_ signal as a function of time (*t*) during recovery was then fitted to a model
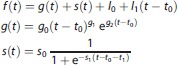
1described previously 18. From the fit results, post exercise time to peak (TTP) and peak amplitudes were derived.

Single-shot ^31^P MRS spectra were processed and fitted (AMARES 30) using jMRUI 31. Resulting PCr and P_i_ signal intensities and pH values were exported. Where absolute values of concentrations were required, values for the resting-state concentrations of [PCr] = 34 mmol/l and [ATP] = 8.2 mmol/l cellular water were used 32. At rest, 85 % of total creatine was assumed to be phosphorylated 33. The PCr recovery time constant (*τ*_PCr recovery_) and the concentration of depleted PCr at the end of exercise ([PCr] _end exercise_) were determined by fitting a single exponential function to the PCr signals.

From these results, the initial PCr recovery rate
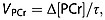
2end exercise ADP concentration
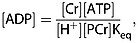
3maximum oxidative phosphorylation according to the linear model
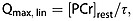
4and the ADP-driven model
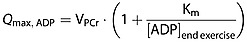
5were derived, as described in 5.

Force and angular position of the pedal were recorded using built-in sensors. The mechanical work was calculated as the time integral over the product of force, angle and lever for each pedal push.

Measured data are given as mean ± standard deviation, if not explicitly stated otherwise. *S*_EPI_(*t*) and pH(*t*) were compared by cross-correlation analysis with a linear model
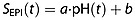
6from *t* = 2 min to *t* = 8 min (i.e. after 1 min of recovery) of the experiment.

## RESULTS

**Figure 1:**
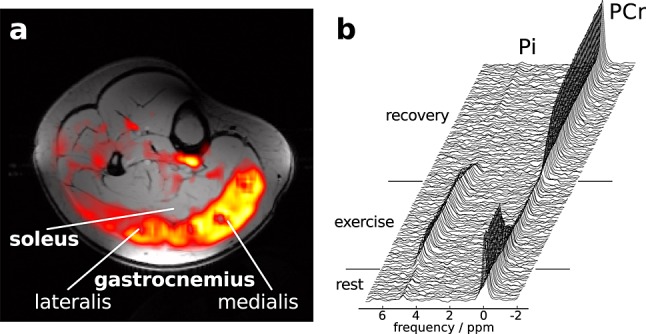
(a) EPI slice of human calf, showing the difference of *S*_EPI_ during the post exercise peak and the pre exercise intensity, overlaid on an axial gradient-echo image. Zero or negative values are transparent. (b) Time series of unaveraged single-shot ^31^P spectra (every 6 s) localised to gastrocnemius medialis.

Muscle energetics derived from localised ^31^P spectroscopy and parameters of *S*_EPI_, measured in two consecutive blocks of a single session, are summarised in Table 1, together with demographic information. 19 subjects completed all measurements successfully, but data quality of two subjects was insufficient due to motion artefacts. High SNR (PCr_rest_: 82 ± 19) resulting from a 7 T scanner and a dedicated, form-fitted RF coil array allowed for tissue-specific placement of the volume of interest in the gastrocnemius medialis. The boundaries between muscle groups were clearly visible in echo-planar images and large vessels were easily identified for exclusion from ROIs. An EPI difference image (recovery - baseline) and a time-series stack of ^31^P spectra are shown in Figure 1. For this purpose, the difference of 30 averaged images during recovery and 20 from baseline were registered to the underlying gradient-echo image (Fig. 1a). The model applied to quantify *S*_EPI_ time courses was found to describe the signal well, with a *R*^2^ of the fit of 0.92 ± 0.13.

**Table 1:** Summary of subject demographics and measured data

	Mean ± standard deviation
Subjects	8 m / 11 f^a^
Age	25.4 ± 2.7 y
BMI	21.5 ± 1.8 kg/m^2^
	
*τ*_PCr recovery_	83 ± 72 s
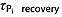	60 ± 45 s
PCr depletion	81 ± 15 %
PCr_end exercise_	6.62 ± 5.08 mmol/l
pH_rest_	7.02 ± 0.03
pH_end exercise_	6.75 ± 0.23
	
TTP *S*_EPI_	178 ± 109 s
Peak post exercise *S*_EPI_	1.24 ± 0.14
Δ*S*_EPI_	0.16 ± 0.08
	
Work / plantar flexion	10.0 ± 2.8 J
Work / area	1.4 ± 0.5 J/cm^2^
Power	3.4 ± 0.9 W
	
ADP	244 ± 199 μmol/l
Q_max,lin_	0.63 ± 0.34 mmol/l/s
Q_max,ADP_	0.55 ± 0.25 mmol/l/s
V_PCr_	0.46 ± 0.19 mmol/l/s

a One female subject was measured twice.

**Figure 2:**
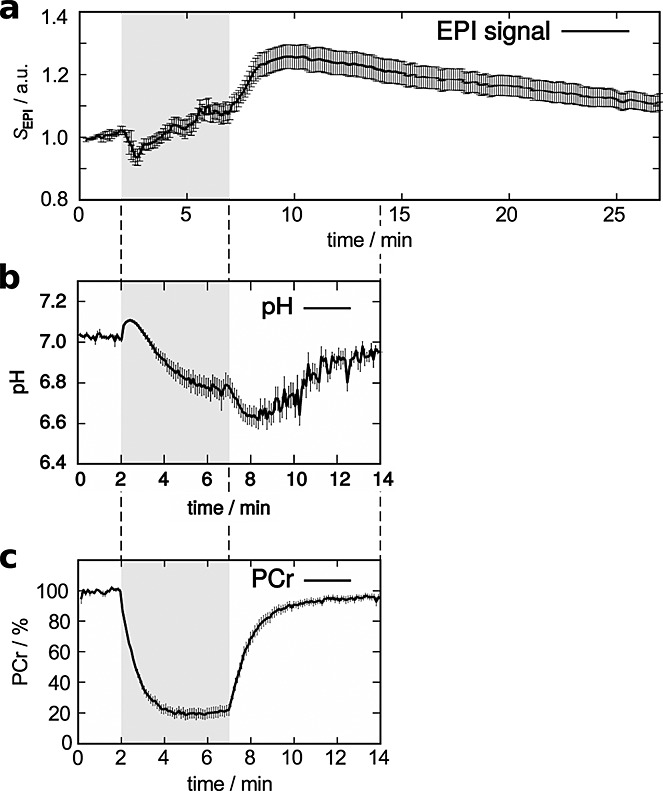
Mean ± standard error of the mean of (a) *S*_EPI_, (b) pH and (c) PCr signal time course. Data from the two subjects with very slow PCr recovery and long TTP *S*_EPI_ were excluded from plots (b and c).

**Figure 3:**
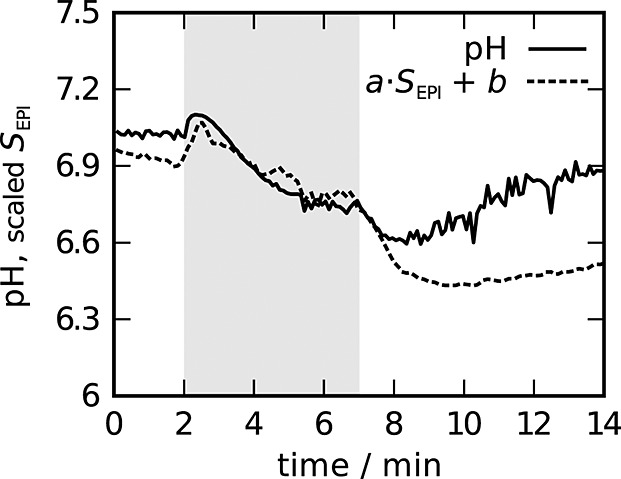
pH(*t*) and rescaled *S*_EPI_(*t*) time courses, mean over all subjects.

During exercise, which started after 2 min of image acquisition at rest, *S*_EPI_ dropped rapidly to a minimum within less than 1 min, before increasing gradually over the remaining exercise period (Fig. 2a). In contrast, pH (Fig. 2b) increased initially before starting to drop. The evolution of *S*_EPI_(*t*) was very similar to the evolution of pH and a linear dependence between *S*_EPI_(*t*) and pH(*t*) was found (cross-correlation coefficient *r* = − 0.85 ± 0.07) from the onset of exercise until 1 min of initial recovery; see Figure 3.

While exercising, gastrocnemius PCr dropped rapidly and reached a steady state after approximately 3 min of exercise (Fig. 2c). After the end of exercise, PCr recovered (Fig. 2c) to pre exercise levels (*τ*_PCr recovery_ = 83 ± 72 s). P_i_ signal dropped faster than PCr increased during recovery (see Table 1); the correlation between the time constants *τ*_PCr recovery_ and 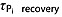 was highly significant (*r* = 0.98, *p* < 10^− 7^). The EPI signal increased rapidly after the exercise and reached its maximum after approximately 3 min post exercise (Table 1, Fig. 2a). In contrast to PCr, *S*_EPI_ did not return completely to pre exercise levels within the duration of the scan (i.e. 27 min).

**Figure 4:**
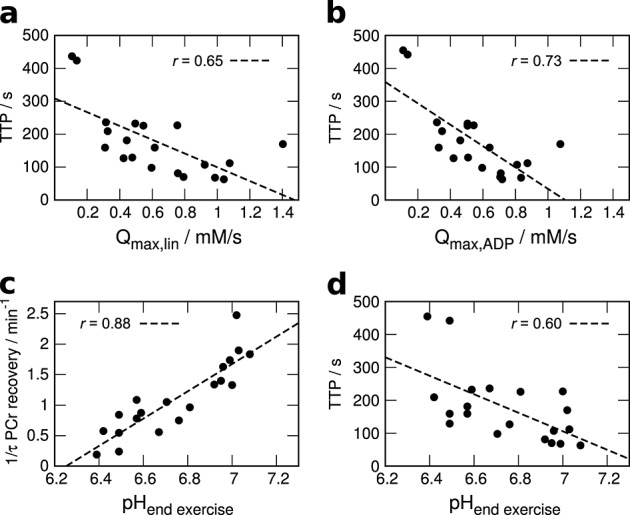
Correlation of TTP *S*_EPI_ and (a) linear and (b) ADP-driven models of maximum oxidative phosphorylation, along with end exercise pH correlated with both (c) PCr recovery rate 1/*τ* and (d) TTP *S*_EPI_.

Recorded force levels were 34 ± 12 % and 38 ± 10 % of individual maximum voluntary contraction force during EPI acquisitions and ^31^P MRS, respectively, and did not correlate significantly with any of the reported parameters, indicating that the intended normalisation of force was effective. Between the two experiments, no significant difference in mechanical work was observed: the inter-block correlation of work per cross-sectional area was (*r* = 0.91, *p* < 10^− 7^). The metabolic response to exercise, however, measured as end exercise PCr depletion, varied considerably between subjects and ranged from 50 to 98 %. PCr depletion correlated significantly with mechanical work divided by the gastrocnemius medialis' cross-sectional area (*r* = 0.50, *p* = 0.02). This heterogeneity in PCr genetive resulted in a reasonable dynamic range of individual data points (see Figs 2c and 4).

**Figure 5:**
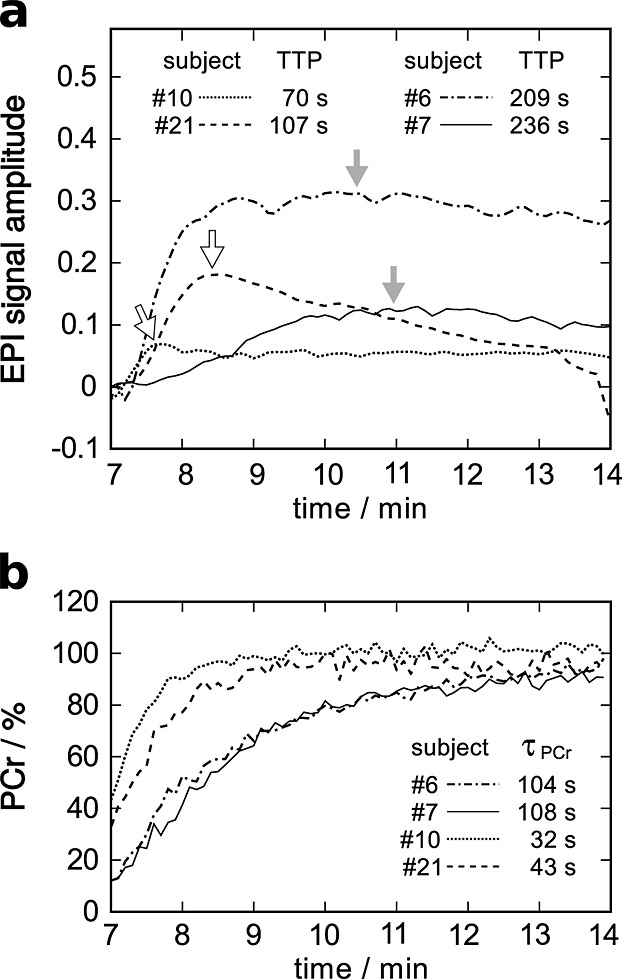
(a) EPI and (b) PCr signal during recovery from exercise of four representative subjects: two with relatively long TTP *S*_EPI_ and two others with short values, as indicated by the arrows and *τ*_PCr recovery_, were selected.

Aside from the correlation of pH(*t*) and *S*_EPI_(*t*), interesting correlations were found between the TTP of the post exercise EPI signal and the time constants of PCr (Fig. 2c) and, likewise, P_i_ recovery. TTP also correlated highly significantly with other measured parameters listed in Table 2, most importantly with both measures of *Q*_*max*_ (Fig. 4a, b). For illustration, the PCr and *S*_EPI_ during recovery of four subjects are shown in Figure 5. Two volunteers with long (*#*6 and *#*7) and two with relatively short (*#*10 and *#*21) *τ*_PCr recovery_ were selected. Subjects with shorter TTP *S*_EPI_ (Fig. 5a) also had shorter *τ*_PCr recovery_ (Fig. 5b). Note that the data shown in Figure 5 are not those from the two subjects with extremely slow PCr recovery and TTP *S*_EPI_ (*#*5 and *#*8). No significant correlations with the time to half-peak value, i.e. how fast *S*_EPI_ returned to baseline, were found.

**Table 2:** Correlation coefficients of TTP *S*_EPI_ and Δ*S*_EPI_ with the most important parameters of ^31^P kinetics and measures of energy metabolism

	*τ*_PCr recovery_	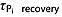	V_PCr_	*Q*_max,lin_	*Q*_max,ADP_	ADP	pH_endex_
TTP *S*_EPI_	0.89^e^	0.89^e^	0.77^d^	0.65^b^	0.73^c^	0.74^c^	0.60^b^
Δ*S*_EPI_	0.41	0.41	-0.47^a^	-0.56^b^	-0.56^b^	0.10	-0.57^b^

a *p* < 0.05

b *p* < 0.01

c *p* < 0.001;

d <10^-4^

e <10^-6^

Also note the correlation between the magnitude of the post exercise 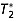 change (Δ*S*_EPI_), defined as peak amplitude minus end exercise *S*_EPI_, and both *Q*_max_ and end exercise pH (see Table 2) when excluding the two subjects (*#*5 and *#*8) with PCr depletion greater than 95 %.

In addition, pH_end   exercise_ correlated with *V*_PCr_ (*r* = 0.75, *p* = 0.0001), 1/*τ*_PCr recovery_ (*r* = 0.86, *p* = 10^− 6^) and PCr depletion (*r* = 0.86, *p* = 10^− 6^).

## DISCUSSION

### pH and 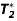 changes

In this study, a strong link between high-energy phosphate and pH kinetics, cellular energy metabolism and the skeletal muscle 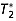 -weighted signal (*S*_EPI_) was observed.

The most interesting result is that, during exercise and the first minute of recovery, the time course of *S*_EPI_ was a linear function of the pH time course (Fig. 3), i.e. cross-correlation analysis showed that pH explained *r*^2^ = 72% of the variance in *S*_EPI_. This result is particularly interesting, given that *S*_EPI_ and pH were measured in consecutive exercise-recovery blocks and not simultaneously. To our knowledge, this has not been shown with high temporal resolution in human muscle before. For the comparison of ^31^P MRS and 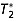 weighted *S*_EPI_, it was essential to achieve the high temporal resolution of 6 s and accurate localisation of both ^31^P and ^1^H data from the same tissue.

Our results confirm earlier findings on the pH dependence of *T*_2_ in healthy volunteers 34 and in frog muscle 35. The underlying mechanism is probably the osmotic pressure change due to intracellular accumulation of metabolites, such as P_i_, Cr or lactate, which drives a proton-dependent water shift between intra- and extra-cellular compartments 12.

After around 1 min post exercise, the P_i_ signal became very small and therefore pH(*t*) quantification was not very reliable. Nonetheless, it is apparent that the linear relationship of *S*_EPI_ and pH does not hold any more during later stages of recovery, i.e. pH returns to baseline values much more rapidly than *S*_EPI_. This, however, is still consistent with the assumption that 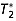 changes are driven by H^+^ levels: when the osmotic gradient across the cellular membrane is lost, only diffusion, which is much slower, remains to restore pre exercise states eventually 2.

### Energy metabolism and 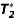 changes

The data presented in this work revealed highly significant correlations of TTP *S*_EPI_ with both measures of *Q*_max_, as well as with *τ*_PCr recovery_. When plotting the data (Figs 2c, 4, 5), the picture is very consistent: more PCr depletion corresponds to lower end-exercise pH, (Fig. 4a) slower PCr recovery 36 and consequently lower *Q*_max_ and, in turn, also a longer time to peak of *S*_EPI_. A plausible and simple explanation is that faster PCr resynthesis, caused by higher mitochondrial ATP production (*Q*_max_) 5, leads to shorter periods of elevated mitochondrial activity and earlier peak acidosis or minimum pH and thus maximum 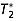 and consequently increased EPI signal.

A correlation analysis of PCr and *Q*_max_ with respect to the amplitude of *ΔS*_EPI_ resulted in significant correlations similar to those of TTP *S*_EPI_, but the correlation coefficients were generally lower.

The actual amount of the contribution of the skeletal muscle BOLD effect to the measured *S*_EPI_ is hard to determine. Obviously there is an initial drop in 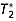 weighted *S*_EPI_, which is not explained by the initial rise in pH; neither is the post exercise *S*_EPI_ maximum. There is a qualitative difference between *T*_2_ and 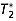 weighted data, in that *T*_2_ apparently increases with the onset of exercise, see for example 13, while 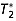 decreased, which can then be attributed to susceptibility effects such as the BOLD effect. At least in frogs 35, skeletal muscle volume changes were found to be another important contribution to changes in *T*_2_.

### Comparison with previous studies

Vandenborne *et al*. 37 used ^31^P Hadamard spectroscopic imaging before and during exercise and 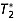 mapping before and immediately following exercise, both with relatively low (∼1 min) temporal resolution. They found significant correlations of 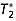 with P_i_ and pH during plantar flexions. In other studies where both ^31^P MRS and *S*_EPI_ were acquired in exercising muscle, the rather large volume of interest was defined based on the sensitive volume of a single loop coil 38,39,28. In our previous work 28, studying ischaemic exercise, *S*_EPI_ and ^31^P spectroscopy were not measured on the same day. All of the above-mentioned studies were performed at static magnetic field strengths between 2 and 4 T.

To our knowledge, a correlation between TTP *S*_EPI_ and PCr recovery has so far only been reported by Wary *et al*. 38. In that study, a correlation between TTP *S*_EPI_ and *τ*_PCr recovery_ was found only in patients with a glycogen storage disorder and not in healthy volunteers. A possible explanation for the discrepancy from our findings, which show strong correlations also in healthy subjects, could be that an exercise intensity based on a target PCr depletion resulted in a smaller dynamic range of the measured parameters compared with our study. Reported TTP *S*_EPI_ values were all shorter than 100 s and *τ*_PCr recovery_ < 70 *s* in the control group, while in this study they ranged up to ∼ 450 and ∼ 300 s, respectively. It should be mentioned that the correlations remain significant even when rejecting these two extreme values. Further differences between this study and the work of Wary *et al*. 38, which could play a role in comparability of the results, are localisation technique, field strength and repetition time used.

In contrast to the strong PCr depletion values measured in this work, a recent study on ^31^P kinetics in the quadriceps muscles with repeated CSI 40 during knee extension exercise found less PCr depletion, even though subjects claimed to be exhausted. The very slow sampling (4 min) using CSI and the definition of true voxel boundaries, limited by the point-spread function, could be at least partially responsible, as the authors themselves discussed 40. Also, because the quadriceps muscles are much bigger than the gastrocnemius, systemic effects could be more limiting than in our study.

### Design and data quality

The ^31^P data acquired in this study were of high quality in terms of line width, SNR and stability over the duration of the experiment. PCr, P_i_ and hence also pH were quantified from single spectra. This applied similarly to 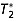 weighted data, as EPI contained few artefacts even during exercise, with the exception of two subjects whose data were not included in the analysis. In an experiment where motion is an essential feature, more motion-related artefacts would have been expected. Fixation in the form-fitted coil and compliance of the subjects were apparently sufficient in 19 of 21 cases. Good data stability was evident from the fact that image registration was not required. An important contribution to ^31^P data quality was the use of a form-fitted calf coil array. The bigger sensitive volume and the more homogeneous *B*_1_ transmit field, compared with a planar loop coil, allowed for voxel placement optimised to the protocol and not limited by the sensitive volume of the coil. The model for the post exercise *S*_EPI_ response fitted the present data as well as previously shown ischaemia-reperfusion data 29.

The authors are aware of the fact that systematic effects of repeated exercise potentially influenced the results and the study design could be improved, but technical limitations and practical considerations, in particular subject compliance and scan time, were considered more important. The excellent agreement between *S*_EPI_ and pH time courses is a strong indicator that no systematic errors were caused by the protocol. No significant differences in muscle force or mechanical workload between the two measurement blocks were found. The observed heterogeneity in PCr depletion and hence a sufficient dynamic range in the measured parameters allowed for a meaningful correlation analysis even in healthy young volunteers.

^31^P data were acquired for 14 min, based on the expected recovery times from the literature 2,4 and previous studies from our lab. *τ*_PCr recovery_ and depletions reported in this study correspond to a PCr resynthesis of > 99 % after 7 min of recovery. On average, pH had not fully recovered to baseline values; however the correlation to *S*_EPI_, reported here, ended much earlier.

^31^P data acquisition was specifically localised to the gastrocnemius medialis muscle. Signals from other tissues, particularly the less working soleus muscle 21, do not contribute, because the contamination from outside the selected VOI with the semi-LASER sequence used was shown to be as low as 1 % 21. Due to *J*-evolution, the ATP signal was too low for reliable quantification; therefore, resting PCr and ATP concentrations were assumed. Figure 1a clearly supports the hypothesis that the main workload was carried by the gastrocnemius muscles only, confirming the validity of the locations of ^31^P MRS voxels and ^1^H MRI ROIs.

Acquiring EPI imaging with long repetition delay and hence low sensitivity to *T*_1_ and inflow effects was an important design feature, allowing us to focus on 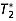 weighting. Actual dynamic 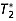 and *T*_2_ parameter mapping would be the next step to investigate.

## CONCLUSIONS

A direct link between mitochondrial function, cellular energy metabolism, acid-base equilibrium and 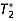 was found during exercise and recovery. In particular, pH time courses apparently induce the observed 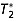 weighted signal time course.

The combination of time-resolved localised ^31^P spectroscopy and 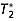 sensitive MRI revealed highly significant correlations between the time constant of PCr resynthesis, the rate of oxidative phosphorylation and the time to peak of the post exercise *S*_EPI_ response. In our opinion, ^31^P studies on exercising muscles benefit significantly from accurate localisation schemes, particularly when conclusions are drawn from comparisons with other localised methods, such as e.g. MR imaging.

This work is an important contribution to the interpretation of the skeletal muscle functional MRI (*S*_EPI_) signal. This is of particular importance when studying pathologies such as diabetes mellitus, where impaired muscle metabolism is known to play an important role in the development of insulin sensitivity and cardiovascular complications are common.

## Abbreviations

ATP: adenosine triphosphate
ADP: adenosine diphosphate
BOLD: blood oxygen level dependent
CSI: chemical shift imaging
EPI: echo planar imaging
MVC: maximum voluntary contraction
PCr: phosphocreatine
P_i_: inorganic phosphate
RF: radio frequency
ROI: region of interest
*Q*_max_: maximum oxidative phosphorylation
semi-LASER: localisation with classical excitation and adiabatic selective refocusing (a single-shot MR spectroscopy sequence relatively insensitive to *B*_1_)
SNR: signal-to-noise ratio
*S*_EPI_: EPI signal intensity
TTP: time to peak
VOI: volume of interest.
